# Vaccination Criteria Based on Factors Influencing COVID-19 Diffusion and Mortality

**DOI:** 10.3390/vaccines8040766

**Published:** 2020-12-15

**Authors:** Ilaria Spassiani, Lorenzo Gubian, Giorgio Palù, Giovanni Sebastiani

**Affiliations:** 1 Istituto Nazionale di Geofisica e Vulcanologia, 00143 Rome, Italy; 2 UOC Sistemi Informativi Azienda Zero—Regione del Veneto, 35131 Padua, Italy; lorenzo.gubian@azero.veneto.it; 3 Department of Molecular Medicine, University of Padua, 35121 Padua, Italy; giorgio.palu@unipd.it; 4 Istituto per le Applicazioni del Calcolo Mauro Picone, Consiglio Nazionale delle Ricerche, 00185 Rome, Italy; giovanni.sebastiani@uniroma1.it; 5 Mathematics Department “Guido Castelnuovo”, Sapienza University of Rome, 00185 Rome, Italy; 6 Department of Mathematics and Statistics, University of Troms∅, N-9037 Troms∅, Norway

**Keywords:** SARS-CoV-2, statistical analysis, vaccines, pandemic preparedness

## Abstract

SARS-CoV-2 is highly contagious, rapidly turned into a pandemic, and is causing a relevant number of critical to severe life-threatening COVID-19 patients. However, robust statistical studies of a large cohort of patients, potentially useful to implement a vaccination campaign, are rare. We analyzed public data of about 19,000 patients for the period 28 February to 15 May 2020 by several mathematical methods. Precisely, we describe the COVID-19 evolution of a number of variables that include age, gender, patient’s care location, and comorbidities. It prompts consideration of special preventive and therapeutic measures for subjects more prone to developing life-threatening conditions while affording quantitative parameters for predicting the effects of an outburst of the pandemic on public health structures and facilities adopted in response. We propose a mathematical way to use these results as a powerful tool to face the pandemic and implement a mass vaccination campaign. This is done by means of priority criteria based on the influence of the considered variables on the probability of both death and infection.

## 1. Introduction

SARS-CoV-2 has rapidly become a pandemic, with sustained human-to-human transmission [1,2] and an exponential rise in the number of COVID-19 cases [3,4]. The reasons for the rapid virus spread include its high transmissibility also from asymptomatic or minimally symptomatic carriers, the apparent absence of cross-protective immunity from related coronavirus infections, as well as the tardy public health response measures [5,6].

Italy was affected early and badly by the pandemic, with the first outbreaks occurring on the 21st of February in Lombardy and Veneto. On the 31st of January, when COVID-19 was still a Public Health Emergency of International Concern, Veneto region had already organized an emergency coronavirus plan. This included, among other measures, an extended diagnostic approach, specially dedicated first aid and hospital wards, along with a biological and clinical register of COVID-19 cases. The plan became active on the 21st of February when two patients from Vo’ village, admitted at the Schiavonia hospital under suspicion of influenza virus infection, were found positive to SARS-CoV-2. On the 23rd of February, following a regional decree, the hospital was rapidly evacuated, and a complete lockdown was imposed on Vo’ village, whose entire population had to undergo swab testing. Meanwhile, other outbreaks were occurring in the Veneto provinces of Padua, Venice, and Treviso and thousands of diagnostic swabs were executed to suspected and at-risk or exposed individuals. A careful monitoring of the contagion wave was conducted, and all data (clinical, epidemiological, and virus-related) were transmitted daily to the information technology service of Veneto region.

The present study deals with data in Veneto, in the period from 28 February to 15 May, a time lapse during which SARS-CoV-2-positive cases were rapidly growing to reach a peak towards 26 March, and then decreasing with lower speed. We stress that 15 May corresponds approximately to the end of the Italian lockdown. The tested population was homogeneously studied for a number of features and events that include gender, age, positivity to test, comorbidities, care location, and death.

The relation among these parameters is of particular biological relevance, since, ideally, it is unbiased from containment and therapeutic measures that were successively adopted to curb the contagion spread. Moreover, the investigation stems from a geographically uniform population in the early stage of the pandemic.

It is clear that the final control of the pandemic requires the development of a harmless and effective vaccine and subsequent massive vaccination campaign. Waiting for this to happen, useful measures to limit the spread of infection and minimizing mortality have been introduced [7]. However, a vaccination campaign over a large population needs months (or even a year) to be implemented. During this period, suitable priority criteria should be designed for the administration of the vaccine, aiming at containing both virus diffusion and mortality. The results of our work on the influence on the infection and mortality of some variables like age and comorbidities can be used to formulate such criteria.

**Figure 1:**
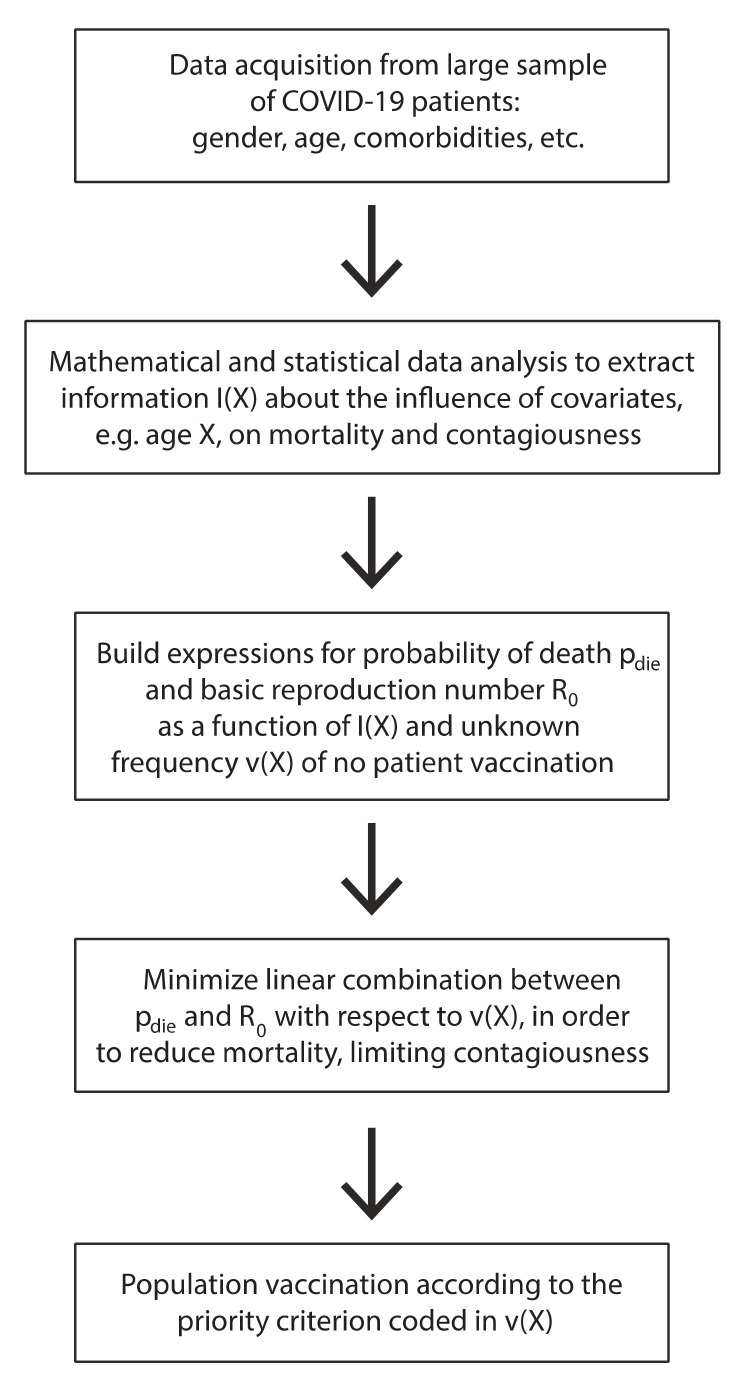
Schematic representation of the work.

The nature of the work described in this paper is explained in the schematic representation given in Figure 1.

## 2. Materials and Methods

The different analyses we conducted are based on robust mathematical and statistical methods and involved two main types of problems: data fitting and hypothesis testing. In both the cases, depending on the specific problem, we adopted either the parametric or the non-parametric approach.

As regards the data fitting, we used the Gaussian kernel density estimation (G-KDE) method for the non-parametric approach [8,9,10], which consists in the linear combination of Gaussian densities centered at observations and with variance depending on a positive parameter, called the bandwidth. The latter’s value was found by an optimal method for the Gaussian kernel. For the parametric approach, we instead used an extended logistic model [11] with an additional parameter, i.e., the exponent of the denominator. In this case, the parameters were optimized following the least squares criterion. Whenever needed, in order to reduce the periodic effect of the weekly varying number of swabs, we considered an additive pure-sine periodic component.

Concerning the hypothesis testing, within the non-parametric statistics, we performed the Kolmogorov–Smirnov (KS) test [10,12] for the null hypothesis of two empirical distributions to be statistically different from each other (e.g., distribution of first symptoms time for males vs. females). The Chi-squared test [10] was instead adopted when dealing with contingency tables as a parametric method to assess the dependence of the probability of a certain event (e.g., death) on a given factor (e.g., comorbidities).

All the methods were implemented by us in MATLAB language. In particular, we developed the code of the KS test and the contingency table Chi-squared test. The MATLAB (MathWorks, Natick, MA, USA) functions *ksdensity* and *nlinfit* were used for Gaussian Kernel density estimation and non-linear fitting of the parametric models to the data, respectively.

## 3. Results

As anticipated in the introduction, we analyzed the COVID-19 data of patients relative to the Veneto region health system (Italy), in the period 28 February–15 May 2020. Data were provided by “UOC Sistemi Informativi Azienda Zero–Regione del Veneto”. Depending on the type of analysis we performed, we received anonymous and aggregated data in Microsoft Excel.

### 3.1. Influence of Age

**Figure 2:**
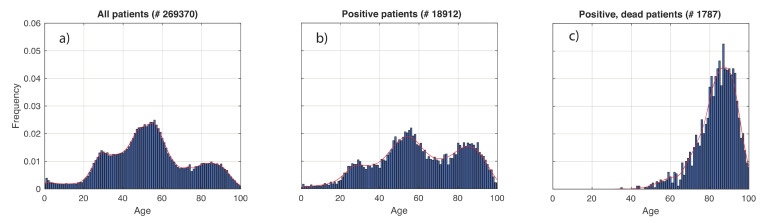
Age distributions of COVID-19 patients relative to the Veneto region health system (Italy), in the period 28 February–15 May 2020. (**a**–**c**) respectively refer to: whole tested patients, positive, and dead ones. The number of patients is also reported. The age range was discretized in equally spaced subintervals of 1 year. Means and standard deviations obtained are respectively: 53.4 and 20.8 for all the patients, 60.3 and 22.0 for the positive ones, and 83.6 and 10.1 for the positive, dead patients.

In Figure 2, we show the results obtained for the age distribution of the whole population and in the subsets of COVID-19 positive and dead patients. Superimposed to the empirical distributions, we also plot the G-KDE. In Figure 2a,b, we observe three peaks. They reflect the demographic profile in Italy, schematically associated to the three main age groups of 0–30, 30–70, and 70–100 years. In fact, 30 is approximately the location of the local minimum between the first and second peaks, while 70 is the same for the second and third peaks. Figure 2b relative to positive patients, shows a statistically significantly different pattern from that in Figure 2a (KS-test: *p*-value < 10^−3^). The relative weights of the three peaks changed, in favor of the oldest ages. We also found statistically significant differences when comparing the positive patients (Figure 2b) to the dead ones (Figure 2c) (KS-test: *p*-value < 10^−3^).

**Figure 3:**
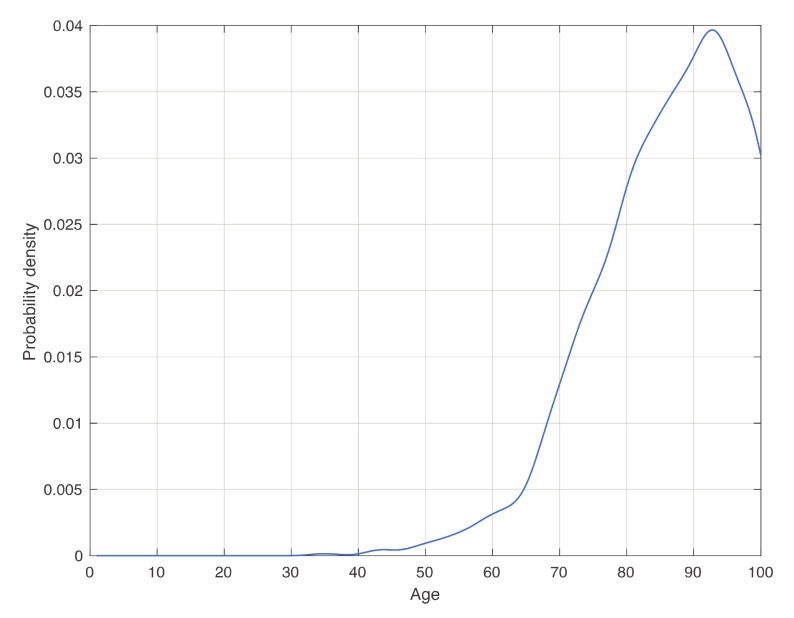
Distribution of dead patients conditioned to the test positivity. To obtain the figure, we took for each age the ratio between the kernel density estimation in Figure 2c by that in Figure 2b, and then we normalized it. The age range was discretized in equally spaced subintervals of 1 year.

In Figure 3, we show the estimate of the death age distribution density, conditioned to test positivity. We observe that the density increases until the peak approximately linearly with three different intervals, identified by the vertical dashed red lines in Figure 3. This could be useful in hospitals to evaluate the death risk of patients. Although intuition would suggest a monotonical increase of this curve, it decreases after approximately 92 years. In fact, the decrease of the density after its peak in Figure 2c is due to the decrease of the frequency of positive patients in the final part of the age range, as observable in Figure 2b. However, when dividing the density in Figure 2c by that in Figure 2b, this pattern should disappear. This does not happen because we likely underestimate the data, and therefore also the tail of the last density.

### 3.2. Influence of Gender

**Figure 4:**
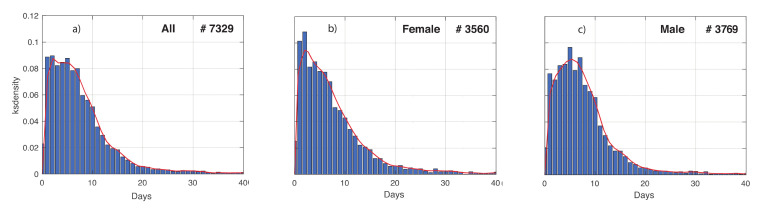
Time between first symptoms and first positive test of COVID-19 patients in Veneto, in the time interval 24 February–15 May 2020. The temporal range was discretized in equally spaced subintervals of 1 day. (**a**–**c**) refers to the positive tested whole population, females and males, respectively. The number of patients is also reported. Means and standard deviations obtained are respectively: 7.89 and 7.28 for all the patients, 7.84 and 7.5 for the females, and 7.93 and 7.06 for the males.

In Figure 4, we show the distribution of the time between first symptoms onset and first positive test, denoted by t_SP_. This can be very useful when adopting informative models to describe the evolution of the COVID-19 epidemic, such as the compartment ones. In fact, this kind of models contains one parameter for the transition rate between the compartment of infected patients developing symptoms, and the one of COVID-19 diagnosis. The reciprocal of the transition rate is proportional to t_SP_; therefore, its distribution can be used in a Bayesian framework as a prior density [13]. The influence of the gender on this distribution was investigated. Based on the empirical distribution, G-KDE was performed to fit the data. The means obtained for the females are shorter than for males. More interestingly, the shape of the two empirical distributions looks different to each other. This difference is statistically significant, in fact the KS-test resulted in a *p*-value < 10^−3^. A further investigation is needed to study the influence of social and biological factors on these results.

By the KS-test, we did not obtain any variation with respect to gender in the distribution of both the time between first positive test and being cured (means and standard deviations for females (males) are 22.13 and 9.56 (22.16 and 9.91)), and the time between first positive test and death (means and standard deviations for females (males) are 12.58 and 11.48 (13.19 and 11.03)).

In Table S1 of the Supplementary Materials, we show the 2 × 2 contingency table for the probability of positivity to COVID-19 conditioned to the gender. The overall probability of all subjects set is 7%. This probability increases to 7.8% for males and decreases to 6.6% for females. Although small, the difference is statistically significant, as shown by a Chi-squared test (*p*-value < 10^−3^). The same pattern is observed when considering the event of death of positive COVID-19 patients in Table S2 of the Supplementary Materials. In this case, the overall probability is 9.4%, increasing to 11% for males and decreasing to 7.9% for females. Additionally, here, the difference is significant (Chi-squared gives a *p*-value < 10^−3^).

### 3.3. Influence of Patient Care Location

The event of death was further analyzed with respect to the location where patients stayed during their disease. First, we considered patients over 60 years old in two conditions: either being in the hospital/medical structure (but not in Intensive Care Unit (ICU)), or in an elderly house. From Table S3 of the Supplementary Materials, we can see that the probability of death is 24% for patients in hospital/medical structures and 14% for those in an elderly house. The difference is statistically significant (Chi-squared test: *p*-value < 10^−3^).

For patients under 60 years old, we instead replaced the elderly house condition by the one of being at home (see Table S4 of the Supplementary Materials). Here, the death probabilities are 1.6% for patients in hospital/medical structures and 0.09% for those at home (Chi-squared test: *p*-value < 10^−3^).

We now move to the case of patients admitted to ICU. As seen in Table S5 of the Supplementary Materials, the probability of death is 38% (8.1%) for patients in (not in) ICU (Chi-squared test: *p*-value < 10^−3^).

The analysis of death probability with respect to ICU admission was also performed in combination with another relevant variable, that is the age of positive patients. For those below 60 years, the probability of death when moving from no ICU to ICU increases here by almost a factor of 60. This is not the case for those above 60 years old, where the increase reduces to a factor of 2–3. These results are shown in Tables S6 and S7 of the Supplementary Material, with both obtaining a *p*-value < 10^−3^ from the Chi-squared test.

### 3.4. Influence of Comorbidities

**Figure 5:**
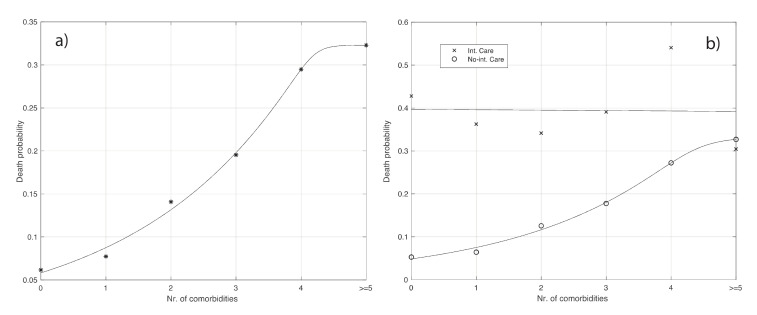
(**a**): Probability of death as a function of the number of comorbidities (see also Table S8 of the Supplementary Materials). The continuous line represents the best fit given by the extended logistic function. (**b**): the same as (a) but relative to being admitted or not to ICU (see Tables S9 and S10 of the Supplementary Materials). For the patients admitted to ICU, the best fit is given by a horizontal straight line. Instead, the best fit for the death probability of patients not being admitted to ICU is obtained with an extended logistic function (continuous curve in the panel).

As seen from the last column in Table S8 of the Supplementary Materials, the death probability increases with the number of comorbidities. This increase is statistically significant (Chi-squared test: *p*-value < 10^−3^). The increase can be even better appreciated by looking at Figure 5a.

We further analyzed the probability of death by considering the combination with a second factor, i.e., admission in ICU. The values of death probability in the last column of Table S9 of the Supplementary Materials are not statistically different (Chi-squared test). Instead, for the case of no ICU admission (see Table S10), the increase of death probability with the number of comorbidities, as seen in the last column, is statistically significant (Chi-squared test: *p*-value < 10^−3^).

The data shown in Tables S9 and S10 are also plotted in Figure 5b. We deduce that the number of comorbidities does not increase the chance of death when people are admitted to ICU. Instead, an increase appears in the case of no ICU.

As seen in the previous subsection, the condition of being admitted to ICU significantly increases the probability of death from 0.3% to 18% for patients under 60 years old (see Table S6). To evaluate if this result depends on the presence of comorbidities, we repeated the analysis by excluding them (see Table S11 of the Supplementary Materials). However, also here, there is an increase from 0.07% to 14% (Chi-squared test: *p*-value < 10^−3^).

## 4. Impact of the Results on Public Health

The present paper is a really comprehensive description of the epidemiological and clinical impact of the COVID-19 pandemic on Veneto region, an area of 18,399 km^2^ with nearly 5 million inhabitants.

The results show that age is playing a critical and statistically significant role in determining the groups of individuals most prone to being infected by SARS-CoV-2 (group over 70 years) and to die from COVID-19 (group over 80 years). While the age peak for death is similar to that reported in the literature [14], the prevalent age of COVID-19 incidence is different from the one that occurred in other European nations like Germany, where young individuals were the most affected in the population [15,16].

The Veneto cohort also shows that the probability of getting infected or dying from SARS-CoV-2 is greater for males than females, a result similar to what is described by others [17]. An X chromosome polymorphism of the Angiotensin Converting Enzyme 2 receptor as well as an androgenic hormone-driven expression of the furin-like transmembrane serine protease 2 have been implied [18,19]. In addition, a male-specific development of anti-interferon antibodies was recently suggested [20]. Moreover, females exhibit a significantly shorter time interval between symptoms and first positive test and no statistically significant difference with males between first positive test, cure, or death, features that are more akin to behavioral than to biological differences.

As expected, COVID-19 patients over 60 have a higher death probability when admitted to ICU with respect to patients under 60. A surprising result is the observation that admission to ICU with respect to no ICU admission carries a higher relative death risk increase (60 fold) for younger patients than for older patients (2–3 fold). This paradoxical phenomenon could rest on the fact that patients over 60 may have been suffering for a longer period from a number of multiorgan pathological conditions that intensive care treatment can alleviate but not stop. Instead, patients under 60 could be in a more acute phase of organ damage due to an abrupt virus systemic infection that dramatically increases fatality.

In agreement with several observations [21], our data also show that the number of comorbidities is directly linked to more severe cases and death. However, this phenomenon does not hold true for patients in ICU, whose clinical status is most likely so serious that comorbidities can have only a minor effect on the chance to live.

We have shown how different clinical and epidemiological variables impacted on COVID-19 evolution at an early stage of the pandemic, under conditions at which medical treatment was not yet optimized due to a lack of properly controlled clinical studies. A number of lessons and insightful thoughts can be drawn from this analysis. First, attention should be payed to prevent contagion spreading to elderly people and subjects suffering from comorbidities, who represent the most exposed and SARS-CoV-2-susceptible populations. They should be carefully protected and isolated. Second, ICUs represent the last resource from which to seek help from, since we have recently shown in different national and regional realities that a direct relation exists between an excess of ICU bed availability and COVID-19 mortality [22]. Third, when dealing with hospitalized patients, antiviral treatment with Remdesivir, the only effective drug against SARS-CoV-2 lower respiratory tract infection [23], should be started early in the symptomatic phase, a common practice for many viral infections. Critical manifestations, such as cytokine storms [24], that necessarily lead to ICU transfer should also be prevented by carefully monitoring markers of inflammation, coagulopathy, alveolar, and endothelial damage [25]; an appropriate use of inhibitors of these processes could be a life-saving procedure pre-ICU.

## 5. Mathematical Implementation of Vaccination

In this section, we describe how to use the results previously obtained to implement a SARS-CoV-2 vaccination campaign. The final goal is to eliminate, or significantly reduce, virus circulation. Since it may take several months or even a year to provide the vaccine to some tens of millions of people in a country, selective criteria should be used to limit mortality in the meanwhile. The results presented above on the influence of the contagiousness and mortality of some covariates like age and number of comorbidities, could be used to formulate such criteria quantitatively within a mathematical framework.

Let us now focus on the age covariate. As we have seen in Figure 2b,c, the probability of being infected and the one of dying significantly depends on age, especially for mortality. Therefore, if we want to limit mortality during the vaccination campaign, priority should be given to older people because of their very high risk of dying. As said before, we also need to limit the spread of the virus to which older people seem to contribute less as being less infected (see Figure 2b) and they have a lower average number of contacts. Hence, this population layer should not be prioritized for vaccination. The two qualitative criteria conflict with each other and a trade-off between them is needed. A mathematical approach could solve this problem by quantitatively accounting for contagiousness and mortality at the same time. Hereafter, we will first focus on preserving lives of the population. Then, we will move to the case of reducing virus circulation. Finally, we will consider both objectives at the same time.

Let us consider the overall probability of death during vaccination, which is given by:

(1) $$p_{die}=\;\int_{0}^{\infty }\varphi_{die|X}\left(X=x\right)\;p_{X}\left(x\right)\;f_{X}\left(x\right)\;v\left(x\right)dx,$$

where $\varphi_{die|X}\left(X=x\right)$ is the probability density function (PDF) of dying from COVID-19 conditioned to the age x of a positive subject, $p_{X}\left(x\right)$ is the probability of being positive to SARS-CoV-2 of an individual given his age x, $f_{X}\left(x\right)$ is the PDF of the age x of a subject in the population considered, while $v\left(x\right)$ is the probability of non being vaccinated for an individual of age x. For the sake of simplicity, we assume here that vaccinated individuals cannot die from COVID-19. The function$\;f_{X}\left(\cdot \right)$ integrate to 1. For practical purposes, we discretize the above equation with the step of one year, obtaining:

(2) $$p_{die}=\;\overset{x_{max}}{\underset{x_{i}\;=\;x_{min}}{\sum }}\varphi_{die|X}\left(X=x_{i}\right)\;p_{X}\left(x_{i}\right)\;f_{X}\left(x_{i}\right)\;v\left(x_{i}\right),$$

with:

(3) $$\overset{x_{max}}{\underset{x_{i}\;=\;x_{min}}{\sum }}f_{X}\left(x_{i}\right)\;=1,\;\overset{x_{max}}{\underset{x_{i}\;=\;x_{min}}{\sum }}f_{X}\left(x_{i}\right)v_{i}=p_{v},$$

where $p_{v}$ is the fraction of population not being vaccinated and $v_{i}=v\left(x_{i}\right),i=1,\ldots ,N$, with N being the number of $x_{i}$ in the COVID-19 death age support [$x_{min},\;\;x_{max}]\;$of the analyzed set of patients. We then have to solve the constrained optimization problem:

(4) $$\left\{\begin{array}{c} \underset{{\left\{v_{i}\right\}}_{i=1,\ldots ,N}}{\mathrm{argmin}}p_{die}=\underset{{\left\{v_{i}\right\}}_{i=1,\ldots ,N}}{\mathrm{argmin}}\overset{N}{\underset{i\;=\;1}{\sum }}\varphi_{die|X}\left(X=x_{i}\right)\;p_{X}\left(x_{i}\right)\;f_{X}\left(x_{i}\right)\;v_{i},\\ under\;constraints:\\ \overset{N}{\underset{i\;=\;1}{\sum }}f_{X}\left(x_{i}\right)v_{i}=p_{v},\;0\leq v_{1},\ldots ,v_{N}\leq 1. \end{array}\right.$$

This kind of problem is known as the *linear programming problem* [26], which can be solved by the simplex method [27]. When applying the proposed methodology to our dataset, the product $\varphi_{die|X}\left(X=x_{i}\right)\;p_{X}\left(x_{i}\right)\;f_{X}\left(x_{i}\right),$ i=1,…,N, corresponds to the empirical histogram in Figure 2c. Intuitively, to minimize $p_{die}$, small values of$\;v\left(x_{i}\right)$ should be associated to large values of $\varphi_{die|X}\left(X=x_{i}\right)\;p_{X}\left(x_{i}\right)\;f_{X}\left(x_{i}\right)$. Here, this product has a peak around 85 years (see Figure 2c), and therefore, around that peak, we should choose small values of the weights $v_{i}$, which corresponds to a high frequency of vaccination. Instead, in a general condition, one can use the estimate of $\varphi_{die|X}\left(X=x_{i}\right)$ shown in Figure 3, while the other two factors should be estimated from data in the specific context considered. Sharp upper and lower bounds for $v_{i}$ can also be introduced.

We turn now to the case where we focus on the virus circulation. To do this, let us consider the basic reproduction number $R_{0}$, which represents the mean value of number of infections generated by a single positive individual. It is natural to assume that $R_{0}$ depends on age, as it is influenced by both transmissibility and the mean number of contacts of an individual, which, in turn, intuitively depend on age. Then, we can write the mean value of $R_{0}$ during vaccination as:

$${\bar{R}}_{0}\;=\;\overset{x_{max}}{\underset{x_{i}\;=\;x_{min}}{\sum }}R_{0}\left(x_{i}\right)\;p_{X}\left(x_{i}\right)\;f_{X}\left(x_{i}\right)\;v\left(x_{i}\right),$$

where the meaning of the last three factors is the same as before, and we directly deal with the discretized version. Following the same calculations, after replacing $\varphi_{die|X}$ by $R_{0}$*,* we end up with a similar linear programming problem.

The final step consists of taking into account mortality and contagiousness at the same time. To deal with this issue, one can consider the linear combination $\lambda {\bar{R}}_{0}+\left(1-\lambda \right)p_{die}$, where 0≤λ≤1. The two extreme choices above correspond respectively to λ=0 and λ=1. The tuning of the value of λ from 0 to 1 will allow variation of the relative weight of contagiousness and mortality on the final vaccination criterion. One way to proceed consists of first assigning priority to either contagiousness or mortality, that is a political issue. For example, let us give priority to mortality. Then, we consider different values of λ, and for each of them, we find the weights $v_{i}\left(\lambda \right),\;i=1,\ldots ,N,$ which minimize the linear combination above. An optimal λ can therefore be chosen as the smallest value, such that the ${\bar{R}}_{0}\;$computed in $v_{i}\left(\lambda \right)\;$will not exceed a given threshold value, fixed on the basis of epidemiological knowledge. We could act similarly for the opposite choice.

The procedure above can also be applied for the number of the comorbidities covariate. More specifically, focusing for example on the probability of death, the expression of $p_{die}$ becomes:

(5) $$p_{die}=\;\overset{N_{c}}{\underset{i\;=\;0}{\sum }}\varphi_{i}\;f_{i}\;v_{i},$$

where $\varphi_{i}$ is the death probability for a patient with a number *i* of comorbidities, $f_{i}$ is the frequency for a patient of the population under study having *i* comorbidities, $v_{i}$ is the frequency by which an individual with *i* comorbidities is not vaccinated, and $N_{c}$ is the maximum number of comorbidities in the set of patients considered. Following the same calculations as before, we end up with the following linear programming problem:

(6) $$\left\{\begin{array}{c} \underset{{\left\{v_{i}\right\}}_{i=0,\ldots ,N_{c}}}{\mathrm{argmin}}\overset{N_{c}}{\underset{i\;=\;0}{\sum }}\varphi_{i}\;f_{i}\;v_{i}\;\\ under\;constraints:\\ \overset{N_{c}}{\underset{i\;=\;0}{\sum }}f_{X}\left(x_{i}\right)v_{i}=p_{v},\;0\leq v_{0},\ldots ,v_{N_{c}}\leq 1. \end{array}\right.$$

## 6. Conclusions

We think our contribution could help health authorities by providing a useful means for prioritizing vaccination in different population layers according to a really quantitative value of the risk linked to SARS-CoV-2 infection and COVID-19 mortality. The choice of whom to vaccinate first will be the relevant issue when different vaccines with diverse efficacy and side effects will be available.

## Supplementary Materials

The following are available online at https://www.mdpi.com/2076-393X/8/4/766/s1, Table S1: 2 × 2 contingency table relative to the influence of gender on the probability of being positive to COVID-19 test. The data show that the latter probability is significantly increased for male (Chi-Square test: *p*-value < 10^−3^), Table S2: 2 × 2 contingency table relative to the influence of gender on the probability of death for patients diagnosed with COVID-19 virus. The data show that the latter probability is significantly increased for male (Chi-Square test: *p*-value < 10^−3^), Table S3: 2 × 2 contingency table relative to the influence on the probability of death of environment where patients are during their disease (COVID-19 virus). The table here concerns not being admitted in ICU and of age over 60. The probability of death is significantly increased for patient being in hospital/medical structure, with respect of being in elderly house (Chi-Square test: *p*-value < 10^−3^); Table S4: 2 × 2 contingency table relative to the influence on the probability of death of environment where patients are during their disease (COVID-19 virus). The table here concerns not being admitted in ICU and of age under 60. The probability of death is significantly increased for patient being in hospital/medical structure, with respect of being at home (Chi-Square test: *p*-value < 10^−3^); Table S5: 2 × 2 contingency table relative to the influence on the probability of death of environment where patients are during their disease (COVID-19 virus). The probability of death is significantly increased by a factor 4–5 for patients being admitted in ICU (Chi-Square test: *p*-value < 10^−3^); Table S6: 2 × 2 contingency table relative to the influence on the probability of death of environment where patients are during their disease (COVID-19 virus). The table here concerns patients of age under 60. The probability of death is significantly increased by a factor ≈ 60 for patients being admitted in ICU (Chi-Square test: *p*-value < 10^−3^); Table S7: 2 × 2 contingency table relative to the influence on the probability of death of environment where patients are during their disease (COVID-19 virus). The table here concerns patients of age over 60. The probability of death is significantly increased by a factor 2–3 for patients being admitted in ICU (Chi-Square test: *p*-value < 10^−3^); Table S8: Contingency table relative to the influence of the number of comorbidities on the probability of death for all COVID-19 diagnosed patients. The increase of probability with the number of comorbidities, as seen in the last column, is statistically significant (Chi-Square test: *p*-value < 10^−3^); Table S9: Contingency table relative to the influence on the probability of death of the number of comorbidities, for COVID-19 diagnosed patients. The table here concerns being admitted in ICU. The increase of probability with the number of comorbidities, as seen in the last column, is statistically non-significant, as obtained by the Chi-Square test; Table S10: Contingency table relative to the influence on the probability of death of the number of comorbidities, for COVID-19 diagnosed patients. The table here concerns not being admitted in ICU. The increase of probability with the number of comorbidities, as seen in the last column, is statistically significant (Chi-Square test: *p*-value < 10^−3^); Table S11: 2 × 2 contingency table relative to the influence on the probability of being admitted in ICU. The table here concerns patients of age under 60, with no comorbidity. The probability of death is significantly increased for patients admitted in ICU (Chi-Square test: *p*-value < 10^−3^).
