# Supplementary material for: Vaccination Criteria Based on Factors Influencing COVID-19 Diffusion and Mortality
Source: Vaccines (Basel). 2020 Dec 15;8(4):766. doi: 10.3390/vaccines8040766 (PMC7765372; doi:10.3390/vaccines8040766)
Supplement: Supplementary file 1 [file vaccines-08-00766-s001.pdf]

Supplementary Materials

Contingency tables.

Tab.S1 2x2 contingency table relative to the influence of gender on the probability of being positive to Covid-19 test. The data show that the latter probability is significantly increased for male (Chi-Square test: p-value<10^{-3}).

\	POSITIVE	NEGATIVE	Tot.	Positive Probability
FEMALE	10773	153180	163953	0.066
MALE	8139	97300	105438	0.078
Tot.	18912	250480	269391	0.07

Tab.S2 2x2 contingency table relative to the influence of gender on the probability of death for patients diagnosed with Covid-19 virus. The data show that the latter probability is significantly increased for male (Chi-Square test: p-value<10^{-3}).

\	DEATH	NO-DEATH	Tot.	Death Probability
FEMALE	857	9916	10773	0.079
MALE	930	7208	8139	0.11
Tot.	1787	17124	18912	0.094

Tab.S3 2x2 contingency table relative to the influence on the probability of death of environment where patients are during their disease (Covid-19 virus). The table here concerns not being admitted in ICU and of age over 60. The probability of death is significantly increased for patient being in hospital/medical structure, with respect of being in elderly house (Chi-Square test: p-value<10^{-3}).

(NO-INTENSIVE CARE, over 60)	DEATH	NO-DEATH	Tot.	Death Probability
HOSPITAL / MEDICAL STRUCT.	659	2097	2756	0.239
ELDERLY HOUSE	350	2175	2525	0.139
Tot.	1009	4272	5281	0.191

Tab.S4 2x2 contingency table relative to the influence on the probability of death of environment where patients are during their disease (Covid-19 virus). The table h**Tab.S5** 2x2 contingency table relative to the influence on the probability of death of environment where patients are during their disease (Covid-19 virus). The probability of death is significantly increased by a factor 4-5 for patients being admitted in ICU (Chi-Square test: p-value<10^{-3}).

(NO-INTENSIVE CARE, under 60)	DEATH	NO-DEATH	Tot.	Death Probability
HOSPITAL / MEDICAL STRUCT.	19	1141	1160	0.016
HOME	8	8514	8522	0.0009
Tot.	27	9655	9682	0.003

Tab.S5 2x2 contingency table relative to the influence on the probability of death of environment where patients are during their disease (Covid-19 virus). The probability of death is significantly increased by a factor 4-5 for patients being admitted in ICU (Chi-Square test: p-value<10^{-3}).

\	DEATH	NO-DEATH	Tot.	Death Probability
INTENSIVE CARE	311	499	810	0.384
NO-INTENSIVE CARE	1476	16626	18102	0.081
Tot.	1787	17125	18912	0.094

Tab.S6 2x2 contingency table relative to the influence on the probability of death of environment where patients are during their disease (Covid-19 virus). The table here concerns patients of age under 60. The probability of death is significantly increased by a factor ≈ 60 for patients being admitted in ICU (Chi-Square test: p-value<10^{-3}).

(under 60)	DEATH	NO-DEATH	Tot.	Death Probability
INTENSIVE CARE	40	184	224	0.178
NO-INTENSIVE CARE	27	9655	9682	0.003
Tot.	67	9839	9906	0.007

Tab.S7 2x2 contingency table relative to the influence on the probability of death of environment where patients are during their disease (Covid-19 virus). The table here concerns patients of age over 60. The probability of death is significantly increased by a factor 2-3 for patients being admitted in ICU (Chi-Square test: p-value<10^{-3}).

(over 60)	DEATH	NO-DEATH	Tot.	Death Probability
INTENSIVE CARE	271	315	586	0.462
NO-INTENSIVE CARE	1449	6971	8420	0.172
Tot.	1720	7286	9006	0.191

Tab.S8 Contingency table relative to the influence of the number of comorbidities on the probability of death for all Covid-19 diagnosed patients. The increase of probability with the number of comorbidities, as seen in the last column, is statistically significant (Chi-Square test: p-value<10^{-3}).

\	DEATH	NO-DEATH	Tot.	Death Probability
NO COMORBIDITY	564	8582	9146	0.062
1 COMORBIDITY	382	4569	4951	0.08
2 COMORBIDITIES	413	2519	2932	0.14
3 COMORBIDITIES	257	1058	1315	0.19
4 COMORBIDITIES	130	311	441	0.29
>=5 COMORBIDITIES	41	86	127	0.32
Tot.	1787	17125	18912	0.094

Tab.S9 Contingency table relative to the influence on the probability of death of the number of comorbidities, for Covid-19 diagnosed patients. The table here concerns being admitted in ICU. The increase of probability with the number of comorbidities, as seen in the last column, is statistically non-significant, as obtained by the Chi-Square test.

(Intensive Care)	DEATH	NO-DEATH	Tot.	Death Probability
NO COMORBIDITY	92	123	215	0.43
1 COMORBIDITY	78	139	217	0.36
2 COMORBIDITIES	71	137	208	0.34
3 COMORBIDITIES	43	67	110	0.39
4 COMORBIDITIES	20	17	37	0.54
>=5 COMORBIDITIES	7	16	23	0.3
Tot.	311	499	810	0.38

Tab.S10 Contingency table relative to the influence on the probability of death of the number of comorbidities, for Covid-19 diagnosed patients. The table here concerns not being admitted in ICU. The increase of probability with the number of comorbidities, as seen in the last column, is statistically significant (Chi-Square test: p-value<10^{-3}).

(NO-Intensive Care)	DEATH	NO-DEATH	Tot.	Death Probability
NO COMORBIDITY	472	8459	8931	0.05
1 COMORBIDITY	304	4430	4734	0.06
2 COMORBIDITIES	342	2382	2724	0.13
3 COMORBIDITIES	214	991	1205	0.18
4 COMORBIDITIES	110	294	404	0.27
>=5 COMORBIDITIES	34	70	104	0.33
Tot.	1476	16626	18102	0.08

Tab.S11 2x2 contingency table relative to the influence on the probability of being admitted in ICU. The table here concerns patients of age under 60, with no comorbidity. The probability of death is significantly increased for patients admitted in ICU (Chi-Square test: p-value<10^{-3}).

(under 60, NO COMORBIDITY)	DEATH	NO-DEATH	Tot.	Death Probability
INTENSIVE CARE	13	77	90	0.14
NO-INTENSIVE CARE	5	6834	6839	0.0007
Tot.	18	6911	6929	0.0026
